# Truck drivers’ perceptions on wearable devices and health promotion: a qualitative study

**DOI:** 10.1186/s12889-016-3323-3

**Published:** 2016-07-30

**Authors:** Rama Greenfield, Ellen Busink, Cybele P. Wong, Eva Riboli-Sasco, Geva Greenfield, Azeem Majeed, Josip Car, Petra A. Wark

**Affiliations:** 1 Global eHealth Unit, Department of Primary Care and Public Health, School of Public Health, Imperial College London, The Reynolds Building, St Dunstan’s Road, London, W6 8RP UK; 2 Health Services and Outcomes Research Programme, Lee Kong Chian School of Medicine, Imperial College & Nanyang Technological University, Singapore, Singapore

**Keywords:** Occupational health, Health promotion, Shift work, Lifestyle, Health technology, Motor vehicles, Public health, Focus groups, qualitative study, mHealth, eHealth, Wearable devices

## Abstract

**Background:**

Professional truck drivers, as other shift workers, have been identified as a high-risk group for various health conditions including cardiovascular disease, obesity, diabetes, sleep apnoea and stress. Mobile health technologies can potentially improve the health and wellbeing of people with a sedentary lifestyle such as truck drivers. Yet, only a few studies on health promotion interventions related to mobile health technologies for truck drivers have been conducted. We aimed to explore professional truck drivers’ views on health promotion delivered via mobile health technologies such as wearable devices.

**Methods:**

We conducted a phenomenological qualitative study, consisting of four semi-structured focus groups with 34 full-time professional truck drivers in the UK. The focus groups were audio-taped, transcribed verbatim and analysed using thematic content analysis. We discussed drivers’ perceptions of their health, lifestyle and work environment, and their past experience and expectations from mobile health technologies.

**Results:**

The participants viewed their lifestyle as unhealthy and were aware of possible consequences. They expressed the need and wish to change their lifestyle, yet perceived it as an inherent, unavoidable outcome of their occupation. Current health improvement initiatives were not always aligned with their working conditions. The participants were generally willing to use mobile health technologies such as wearable devices, as a preventive measure to avoid prospect morbidity, particularly cardiovascular diseases. They were ambivalent about privacy and the risk of their employer’s monitoring their clinical data.

**Conclusions:**

Wearable devices may offer new possibilities for improving the health and wellbeing of truck drivers. Drivers were aware of their unhealthy lifestyle. They were interested in changing their lifestyle and health. Drivers raised concerns regarding being continuously monitored by their employer. Health improvement initiatives should be aligned with the unique working conditions of truck drivers. Future research is needed to examine the impact of wearable devices on improving the health and wellbeing of professional drivers.

## Background

In 2014, there were over 2 million professional drivers in the United Kingdom (UK), of which 53.7 % were road transport drivers or heavy goods vehicle (HGV) drivers. Truck drivers make up 1.6 % of the UK population [1]. Professional drivers, particularly truck drivers, tend to live an unhealthy lifestyle and have increased cardiometabolic risk factors in comparison to other professions [2–5]. Their working conditions are dictated by the global economy that requires industries to operate 24/7 under tight schedules. The irregular working schedule brings along challenges to adopting a lifestyle change [6, 7] because it disrupts healthy eating habits as well as sleep, exercise and social life [8]. Their long and irregular working hours are associated with sleep deprivation which leads to drowsiness, irritability, confusion, as well as impaired attention, recall, reaction time, hand-eye coordination and vigilance level [9]. Fatigue in general is a major risk for driving safety of truck drivers [10]. Their sedentary work style and being constantly on the road allows fewer opportunities to exercise [11] and access to healthy food and maintain a balanced diet, which substantially influence drivers’ wellbeing and performance at work [12, 13]. Truck drivers are also hard to reach for health promotion interventions as they spend long hours driving on the road.

Whereas truck drivers in various countries have different working conditions and each country has different regulations, findings regarding health of truck drivers are rather universal; most of them are obese, work overtime and do not get enough sleep. A survey among American commercial drivers (n = 260) found that the majority (64 %) of them were obese or morbidly obese and at high or very high cardiometabolic risk (89 %). Most of them worked an irregular daily schedule and almost half of them did not get proper night sleep. The more hours they worked daily, the greater was their body mass index. Those who did more shift work and slept less hours had worse sleep quality [14]. Obesity and smoking are twice as prevalent in long-haul truck drivers as in the adult American working population [15]. Over 61 % reported having at least two of the following risk factors: hypertension, obesity, smoking, high cholesterol, limited physical activity, six or fewer hours of sleep per 24-h period [15]. Taiwanese truck drivers who did not get a quality sleep, drove larger yearly distances and drove late at night tended to commit more speeding offenses [16]. UK data suggest that 10–25 % of truck crashes are related to driver fatigue [17]. A survey of 996 UK HGV drivers reported that drivers who snore whilst sleeping at night, were more likely to be obese, had a noticeably large collar size, and had higher accident liabilities than those not reporting these characteristics. Drivers who tended to report daytime sleepiness had increased accident liability [18]. In another study, 81 % of 192 UK truck drivers reported some musculoskeletal pain during the previous 12 months and 60 % reported low back pain [19].

### Health and safety implications of truck drivers’ impaired health

Beyond the health implications, there are safety and economic implications to truck drivers’ morbidity. Commercial driving and long driving hours are leading causes of fatal work injuries, accounting for 40 % of all-cause fatal occupational injuries in America [20], and 17 % of fatal accidents in the EU [21]. Such accidents are commonly linked to human error caused by the truck driver [22].

Current regulations in the UK, EU and US state clear restrictions on driving hours limits (Table 1). However, in the current reality of the haulage industry, drivers often work beyond these limits [23]. Drivers may be also required to undertake shift work, causing disruption to their daily working and sleeping times [14]. Indeed, studies [24, 25] indicated that long-haul truck drivers do not obtain enough sleep required for alertness on the job. For example, for approximately 13 % of the long-haul drivers (n = 184), the mean driving time per shift exceeded the EU regulation, and about 40 % of the long-haul drivers and 21 % of the short-haul drivers reported having problems in staying alert on at least 20 % of their drives [24].

**Table 1:** Driving safety regulations in the UK, EU and the US

UK [43]	
In any working day the maximum amount of driving permitted is 10 h	
In any working day the maximum amount of duty permitted is 11 h. A driver is exempt from the daily duty limit (11 h) on any working day when a driver does not drive.	
EU [44]	
A maximum amount of daily driving time of 9 h that can be extended to 10 h no more than twice a week.	
A maximum amount of weekly driving time of 56 h.	
A maximum total accumulated driving time during any two consecutive weeks of 90 h.	
After driving for a period of 4.5 h, a driver must take an uninterrupted break of not less than 45 min, unless he takes a rest period.	
A minimum daily rest of 11 h, which can be reduced to 9 h, no more than 3 times a week.	
A regular weekly rest period of minimum 45 h and a reduced weekly rest period of a minimum of 24 h.	
US [45]	
May drive a maximum of 11 h after 10 consecutive hours off duty.	
May not drive beyond the 14th consecutive hour after coming on duty, following 10 consecutive hours off duty. Off-duty time does not extend the 14-h period.	
May drive only if 8 h or less have passed since end of driver’s last off-duty	
May drive only if 8 h or less have passed since end of driver’s last off-duty or sleeper berth period of at least 30 min.	
May not drive after 60/70 h on duty in 7/8 consecutive days. A driver may restart a 7/8 consecutive day period after taking 34 or more consecutive hours off duty.	

### Workplace health, disease prevention and wellness programmes

There is growing interest in workplace health, disease prevention and wellness programmes to improve health outcomes and productivity and yield lower costs. Such programmes showed return on investment which suggests a wider adoption of such programmes [26, 27]. Programmes associated with favourable outcomes have several characteristics in common including corporate culture, employees and leadership commitment, a participation-friendly corporate policy and physical environment, adaptation to the changing needs of the employees, support of community health organizations, and utilization of technology to facilitate health risk assessments and wellness education [27]. Successful programmes were also well-designed, well-executed, and were evidence-based [28]. However, as yet, employee participation in such programmes is limited in low-wage industries [29, 30]. Health and wellness programmes in trucking companies commonly fall short, and are insufficient to improve health outcomes in a sustainable way [31]. Lemke et al. [31] proposed a systems-based paradigm as a conceptual and methodological framework with the potential to meaningfully advance interventions in blue-collar work settings.

### The role of mobile health in health promotion for truck drivers

Mobile health (mHealth) technologies offer potential solutions to promote a healthier lifestyle for truck drivers. There are various definitions of mHealth [32]. However there is general agreement that it refers to the usage of mobile and wireless devices to deliver remote healthcare and preventative services. Wearable devices include electronic monitors that contain sensors, storage systems or capacity to track individuals’ health behaviours and physical activities. Therefore wearable devices may serve as one channel of mHealth – through integration of mobile technology to record, track and monitor personal health-related information.

The development and availability of mHealth technologies such as smartphones and smart watches enables ubiquitous access to health promotion tools such as reminders and follow-up of biomedical measures. However, studies have noted the negative effects of in-vehicle visual displays in distracting drivers’ attention, and the information overload caused by performing secondary tasks while driving [33].

### Goals

Despite their notable unhealthy lifestyle and increased risk of impaired health, only a few studies on health promotion interventions in truck drivers exist [2–5]. It is unclear how drivers perceive their own health and lifestyle, and how do they make use of such technologies aiming to improve their health. The aim of this study is to explore professional drivers’ perceptions of health promotion and mobile health (mHealth) technological interventions that aim to promote their health and wellbeing. Hence there is a need for health promotion initiatives to improve the health and wellbeing of professional truck drivers which will potentially benefit drivers, employers, other road users and the public health system.

## Methods

### Study design

We conducted a phenomenological, qualitative study to explore the needs and attitudes of professional drivers regarding health promotion interventions, and particularly their perception about the role of wearable health devices in improving their health. We conducted semi-structured focus groups with professional truck drivers. A semi-structured protocol is systematic yet sensitive to the dynamics of the conversation. Focus groups have been found especially valuable for testing new programmes and ideas [34, 35]. Phenomenological research aims to describe a lived experience, and hence a suitable approach for the study question. For Creswell [36], “a phenomenological study describes the meaning for several individuals of their lived experiences of a concept or a phenomenon. The basic purpose of phenomenology is to reduce individual experiences with a phenomenon to a description of the universal essence”. Hence it was more appropriate than other qualitative approaches such as narrative research, grounded theory, ethnography, and case studies. We followed the psychological phenomenology approach [36], which focuses less on hermeneutics (the researcher’s interpretations) and more on a description of the participants’ experience. The procedure of this approach consists of identifying a phenomenon to study, bracketing out one’s experiences, and collecting data from several persons who have experienced the phenomenon, followed by data analysis aimed at reducing the information to significant statements or quotes and combines the statements into themes [37]. We followed the COREQ checklist for reporting findings of interviews and focus groups [38].

### Recruitment and participants

We recruited participants by approaching companies, advertising in truck magazines and using social media such as truck drivers’ blogs. Because directly approaching truck drivers did not result in a sufficiently large sample, we contacted 95 companies, which were mainly UK haulage companies and major retailers. Most of them did not respond or were reluctant to participate. One medium-size logistics company agreed to partake. Posters were supplied to the company, and interested drivers were provided with information sheets. All participants were 18 years or older and able to speak and read English. To become a lorry driver in the UK, one needs to have a full car licence, be over 18 (with some exceptions), and get a professional driving qualification (Certificate of Professional Competence) [39]. All drivers drove only within the UK. We interviewed a purposive sample of 34 participants who participated in one of four focus groups. Although women were eligible, only male drivers participated, which is likely because over 96 % of UK truck drivers are male [1]. The study was approved by the Imperial College Research Ethics Committee (Ref: 14IC2246). The participants were compensated by £20 vouchers upon the completion of the interview. The participants deliberately chose to participate in the focus groups. None have refused to participate or dropped out.

### Data collection

After studying the relevant literature, we defined four themes for discussion, covering prior experiences with health technology, motivations for using health technology, expectations from health technology platforms and wearable devices and views on employers’ health promotion activities (See Appendix 1). The focus groups were designed to fit a period of 90 min. At the beginning of the focus groups, we administered a brief self-reported questionnaire to collect demographic data and self-reported health and lifestyle ratings. No relationship was established with the participants prior to study commencement.

We conducted four focus groups consisting of 5–12 participants each, and lasted between 50 and 60 min. They took place between November and December 2014 at the employer’s premises. Two researchers experienced in moderating group discussions led the focus groups; RG as the lead moderator and EB as a facilitator, who also recorded the nonverbal communication. Both researchers are research assistants trained in qualitative research methods, have a MA degree, and are female. The participants were only informed that the researchers are research assistants at Imperial College London.

To engage positively and gain the trust of the participants, we emphasized that there were no ‘right’ or ‘wrong’ answers, and participants were free to leave at any time. We emphasized that individual responses will be kept confidential, allowing them to freely express their views. During the focus groups we used clinical interview techniques, such as reflection, restatement, clarification, and exploration. There was no one else present in the room besides the participants and researchers. The discussions were audio-taped and later transcribed verbatim by RG and EB, and were verified by CPW. Any identifying details were removed during transcription. The participants’ names were kept confidential, and the transcriptions were not shared with the management of the company.

### Data analysis and thematisation

The aim of the data analysis within the empirical phenomenology approach, is to reduce the information to significant statements or quotes and to combine the statements into themes [37]. Using a thematic content analysis is useful for this purpose. The analysis was based on the transcriptions of the audio recordings as well as on notes taken of nonverbal communication of the participants. The open coding was done by two qualitative researchers (RG, EB) who independently analysed the data and generated initial codes. We developed the codes for each interview as a standalone unit and then combined them into a mutual set. We developed the codes and themes in an iterative process where codes led to shaping broader themes and vice versa. The meaning of the iterative process is working in a data-codes-themes cycles, where initial codes (i.e., repetitive matters) are first drawn from the text and serve as building blocks for higher level themes. The new themes are then tested against further data, and so on, until the codes and themes system make sense. The themes were derived from the raw data, yet some of them followed the themes predefined in the protocol. We conducted ongoing discussions with the other authors on coding and interpretation of the data. Coding and thematisation continued until thematic saturation was reached. Because the participants were employees of the commercial company and work under tight schedules, the transcripts weren’t returned to participants for comments nor were the participants asked to provide feedback on the findings.

## Results

The drivers were 42.2 ± 7.8 years old on average, and on average had been in the profession for 16.1 years. All of them were male, and most of them drove a HGV at long distance. Most of them perceived their health as “good” and their lifestyle as “healthy” (Table 2). We identified five themes interlacing truck drivers’ perceptions of wearable health technology devices. The themes refer to barriers to maintaining a healthy lifestyle, wearable devices as a means to improve health and wellbeing, motivation for using wearable devices, the experience of being continuously monitored.

**Table 2:** Descriptive characteristics of the study participants

Characteristic	Statistics
Age (years), mean ± SD; range	42.2 ± 7.8; [27–57]
Sex, n (% male)	34 (100)
Driving experience (years), mean ± SD; range	16.1 ± 9.9; [1–33]
Vehicle type, n (%)	
Long-haul HGV^a^	20 (58.8)
Short-haul HGV^a^	7 (20.6)
Both long-haul and short-haul	7 (20.6)
Perception of health, n (%)	
Fair	2 (5.9)
Good	20 (58.8)
Very good	12 (35.3)
Perception of lifestyle, n (%)	
Not very healthy	2 (5.9)
Fair	14 (41.2)
Healthy	16 (47.1)
Very healthy	2 (5.9)

^a^Heavy Goods Vehicle

### Barriers to maintaining healthy lifestyle: the gap between awareness and reality

The first theme relates to the gap between the drivers’ awareness of benefits of having a healthier lifestyle and the reality of living an unhealthy lifestyle. Most of the drivers acknowledged that they live an unhealthy lifestyle, in terms of unbalanced diet and inadequate exercise. Many of them mentioned they do not sleep the amount of needed hours before starting their shift. Yet these habits were perceived as an inherent, unavoidable consequence of their occupation. When we asked about their work environment and how it could be integrated with a healthier lifestyle, the drivers described that “stress”, “long shifts” and “tight deadlines” withhold them from maintaining a healthy lifestyle. For example, some drivers mentioned that their tight schedule and spending long hours away from home limit their opportunities to visit health professionals. However, they acknowledged that not visiting health professionals may put them at risk. For example:

*“We know it’s unhealthy for us but you’re stopping at greasy cafes and places at the moment because it’s the first thing you can eat”.*

*“I think if you’d monitor our sleeping patterns you would be amazed! You would be absolutely amazed, some days we only get 3 h of sleep and then go to a 12 h shift.”*

*“We now have the gym downstairs, but after working 12–15 h you won’t say, oh yes I need to go to the gym really.”*

Acknowledging their unhealthy lifestyle, they were keen to gain more knowledge, information and assistance with improving their health and wellbeing. They showed a vast interest in health promotion and said they would like to know more information on various health aspects such as healthy diet and recommended amount of hours of sleep:

*“I want to change my lifestyle, I would like to take more care of my health and wellbeing but in the current work environment it is hard to make a change in my lifestyle”.*

*“It’s just getting the time and the education to change that, to change my lifestyle.”*

### Wearable devices as a means to close the gap between awareness and reality

Following the discussion on their interest to change their unhealthily lifestyle and how their work conditions refrain them from doing so, we discussed the potential role of wearable health devices in helping them improve their health. About a third of the drivers mentioned that they already used mobile health technology devices (mostly apps on their smartphones), but most of them had low or no experience with wearable health technology devices, and were unaware of what kind of wearable health devices are available and which ones are most suitable for their needs. The majority of them were keen to use such technologies:

*“Don’t know what’s out there what I can use, because I don’t mind using it, because I want to improve my health and I understand it is going to be hard work”.*

*“I am interested in how it works what it can tell you and how to obtain it? And obviously if it helps it will be a worthwhile investment”.*

*“It shouldn’t be a quick fix, it should be something that really changes the perception of people’s lifestyle, not just a quick diet.”*

When asked what they would expect from such a device, the drivers highlighted the need to have simple, straightforward functions, which should be customized according to their lifestyle:

*“But if it would involve a lot, it’s going to last about two days. You know what I mean because at the end of the week you’re tired and full of stress… we might stop using it”.*

*“Also the things that are available on the market right now take a regular 9–5 job person into account, but not a truck driver who usually has to go to bed at 5 in the afternoon and get up at 2 in the morning.”*

### Motivation for using wearable devices

We identified two sources of motivation for truck drivers for using wearable devices. The first one was positive motivation based on the potential of wearable devices to help prevent undesired health conditions. They referred them as that will help them to take more responsibility of their health and wellbeing and hence preventing prospective health conditions. They particularly referred to the ability of such devices to continuously monitor their condition and indicate prospect deterioration:

*“Rather than putting their head in the sand, use a piece of technology or telling you from day to day minute to minute how their lifestyle is. And if you changed it you can see it rather than being told or putting your head in the sand”.*

*“Well ok, but I mean incorporating it into something like, it should have a sort of dietary aspect to it you know what I mean, what to eat what not to eat. I’m still sort of on the preventive side of things if you know what I mean”.*

*“I’m just saying, if you went to your doctor …and someone could wear that for a week to monitor. They don’t have to put you on medication, but can see where you’re standing and prevent it. Medicine is moving anyway to preventive medicine, isn’t it?”*

Other drivers wanted to make use such monitoring devices because they were fearful of developing health conditions. They felt that changes or deterioration of their health conditions could be detected by such devices. Recent fatal events (heart attacks) of colleagues who did not have any known health issues, raised the drivers’ awareness and eagerness to partake in prevention initiatives. They said that having a wearable device may give them the confidence and reassurance that if something will happen, particularly heart attacks, they will be able to prevent it at an earlier stage. This motivation is very similar to the first one, there was a slight difference between the two types of motivation: while the first emerged from more positive wish to engage in healthier lifestyle, the second one emerged from fear of developing diseases and unfortunate fatal events:

*“Or when you know someone, like one of my best mates died of a heart attack and he was a HGV driver and that gave me a wake-up call“*

*“Heart attack…. Because if that thing happen it shocks you really. I don’t like it, but if I would have a heart problem or would be diabetic I would probably wear one, because then I have to know what is going on.”*

### The “Big brother”- the experience of being continuously monitored

Possibly due to their work conditions whom they described as ‘lonely’, the drivers felt reassured to have a device that will be constantly with them and will monitor their health, either as a preventive measure, or out of fear of developing diseases. Another aspect of being continuously monitored is their personal data including health conditions being available to their employer. They perceived mHealth monitoring as a way to improve the drivers’ work conditions, as the management will have a better view on drivers’ work performance:

*“Talking about a perspective from the health of a company it gives the management a better idea if they saw what you are actually doing physically and mentally each day. Over the hours or period that you are working“.*

*“If that’s monitoring your heart rate, your blood pressure and your cholesterol level what’s wrong with the company knowing that?*

Sharing personal data not only provides opportunities for employees to receive feedbacks from health professionals and managers, but also allowing company to build an image on caring their employees’ health. Several drivers described their experience of being occasionally put off by their managers. Since sharing drivers’ data with employers may provide indicators on their employees’ health, drivers’ work performance can also come under scrutiny. Hence, the data can provide evidence on performance, which may or may not protect drivers from being judged unfairly:

*“I think at the end of the day you can go to your line manager or your boss or anything like that and they tell you a load of crap like you’re not doing that you’re not doing that mate. You can go, hang on a second, there’s your proof in your pudding. That’s my week at work, that’s when I was off, there’s your blood pressure everything sleep pattern, fine, that’s for two or three weeks go on and compare“.*

The reassurance and benefits of being monitored come at the expense of data privacy and concerns about who will have access to the data collected from the wearable device. The majority of the drivers were worried about risking their jobs if the information was shared with their employers:

*“Yeah well, the sleeping patterns obviously because if your employer could somehow monitor it and your employer can see you only had two hours of sleep and sends you out to a twelve hour journey to London. You know what I mean? It can risk your job”.*

*“Let’s say they can see you are stress and tired, do you really want them to see it?”*

### Who will benefit from wearable devices?

Another theme that arose from the participants’ accounts was the question of who would benefit from using wearable devices. Several drivers were not sure whether having a health monitoring device at work would be merely of economic interest to the organisation, or whether having health monitoring wearable devices would create a mutual benefit for both the employers and the drivers. Several participants referred to the company’s health promotion programme that was launched after the recent fatal events, as a positive change aiming to reduce their health risks and improve wellbeing. For example:

*“I haven’t worked for anyone else who puts me on this much stuff about health and wellbeing”.*

*“The company is doing more with health that it starts bringing it to the top of the chain”.*

However, other drivers were suspicious about the intentions behind the organisation’s efforts (i.e., whether they authentically care for the drivers or motivated by sheer economic interests). They thought that the organisation’s investment in the truck drivers’ health was also related to the occurrence of several cardiovascular related deaths occurred. Therefore the drivers wondered if the company just took a pragmatic approach to sustain its workforce:

*“They want people to work until they retire. It will save them money in the end”.*

*“There is no young coming in, so they now have to look into keeping us lot healthy, because they want us to work until we retire and that’s why they are going to bother”.*

## Discussion

### Summary of findings

The drivers’ accounts raised two main narratives. The first narrative was how drivers relate to their unhealthy lifestyle and the occupational factors refraining them from adopting a healthier lifestyle. The other narrative was the role of mHealth wearable devices in promoting their health while help in prevention health conditions and especially cardiovascular disease. Drivers were aware of their unhealthy occupational lifestyle and its possible consequences. Participants were generally welcoming towards the discussed mHealth devices, yet the introduction of such technologies raised concerns about violation of their privacy and their data being available to their employers as it imposes risk to their jobs. Second, there was some ambivalence towards the interest of companies to provide such devices to their drivers (i.e., whether they authentically care for the drivers or motivated by sheer economic interests).

There was a mismatch between the drivers’ self-rating of health (mostly “good”) and lifestyle (mostly “healthy”) in the questionnaire administered preceding the focus groups, and what they expressed during the focus groups. This may be a result of a cognitive bias (i.e. people report what is easier for them to report, because it is emotionally more difficult to report that one’s health is not so good). Methodologically, it may also serve as an example of the power of qualitative research in bringing up above the surface issues which are difficult to capture in surveys.

### Challenges raised by the findings

#### The importance seen by companies to offer health promotion programmes

The fact that only one out of 95 companies approached was willing to partake in the study is a meaningful finding by itself. While commercial confidentiality, time constraints and lack of direct benefit to the company are likely to have played a role here, it may hint at the relative importance given to looking after drivers’ health and wellbeing by companies. The scarcity of scholarly literature on health promotion interventions for truck drivers as high-risk population may be another echo of the current lack of policy and academic interests in this population. Bearing in mind that in the EU, 46 % of goods transport is transferred via roads (in comparison to 30 % in the USA, 60 % in Japan and 11 % in China [40]), the health of truck drivers is a crucial issue from both public health and economic perspectives. In Europe, including the UK, the shortage of truck drivers is already ‘a ticking bomb’ which may create a logistic bottleneck to the economy [9]. Another challenge here is that truck drivers are hard to reach for health promotion interventions as they are mostly on the road.

Helping employers to reduce road related collisions at work, including through improved HGV safety is one of the UK Government key priorities for road safety [41]. Highways England (a government-owned company with responsibility for managing the core road network in England) aims to move towards its target of reducing the number of people killed or seriously injured on the strategic road network by 40 % by the end of 2020.

#### Tailoring health promotion programmes to drivers’ needs

Another important matter apparent in the data is the need to fully understand the particular needs of the targeted populations to design appropriate public health intervention. In this specific company, efforts have been made to promote health and wellbeing, such as a free gym, a health check machine and healthy snacks in the cafeteria. However, many of these goodwill efforts do not seem to reach the drivers due to their work conditions: their long working hours do not leave them enough time to go to the gym, and being mostly on the road they do not take the full advantage of the cafeteria. To improve the chances of health improvement initiatives are taken by truck drivers, the initiatives should be tailored to their particular work conditions.

#### Compliance with regulatory work hours

A worrying fact is that many of the drivers do not seem to adhere to rest hour regulations, and appear to be afraid of the consequences if this information will be known to their employer. Non-adherence to rest hour regulations may have severe consequences not only on the drivers’ health, but also impinge on the safety of other road users. Several studies [24, 25] indicated that long-haul truck drivers do not obtain enough sleep required for alertness on the job. Adhering to the work hours regulations, access to physical activity facilities and healthy food, as well as improve the ease of access to health professionals, social support are needed to improve the health and wellbeing of truck drivers.

#### The ‘big brother’ issue

Growing number of employers are now using vehicle telematics to monitor their drivers’ location and behaviour. Drivers may be naturally concerned about their driving behaviour monitored by their employer, and who can then access that data and for what purpose. Such data is protected under the Data Protection Act (1998), yet drivers may not be aware of their rights. In the UK, the Royal Society for the Prevention of Accidents produced a policy paper discussing road safety and in-vehicle monitoring technology (driving telematics) [42]. It is mandatory for Event Data Recorders to be fitted in cars and light vehicles sold in the USA from September 2013. In Europe, such technologies became mandatory on all new vehicles in 2015 [42].

### Strengths and limitations

This is the first study that explored professional drivers’ perceptions on mHealth technologies in promoting their health and wellbeing. Methodologically, qualitative data collected in the four focus groups enabled us to gain insights on personal daily experience and the work environment of professional drivers. In addition, the open discussions highlighted drivers’ self-awareness to their unhealthy lifestyle and how drivers’ lifestyle could be influenced by their work environment. However, this exploratory study has several limitations. First, all participants were recruited from the same organisation which may limit the generalizability of our findings and introduced volunteer bias. Yet, qualitative research rarely seeks to generalize but to explore perceptions, ideas and opinions. Second, the particular organisation that was willing to participate was already interested in health promotion, and may not be representative of the haulage industry. Third, it is difficult for people to comment on a concept or on a service that they did not use before. While conducting the interviews we have noticed different levels of knowledge and experience with technology, hence responses were likely based on previous experience with available technologies such as smartphones or pedometers.

### Future research

Future research could examine the impact and experience of using wearable devices, and to understand the impact of health monitoring on driving performance and safety, and drivers’ health and wellbeing. Future studies should strive to include participants from different organisations, including private companies and the public sector. Another research avenue is the employer’s angle, capturing motivations and challenges in promoting health of professional drivers, and their opinions regarding the usage of wearable devices.

## Conclusions

Truck drivers are a high-risk population for various health conditions, and yet a neglected profession from a public health perceptive. The transport sector, the haulage industry and the public health sector should acknowledge these challenges, and mutual actions should be taken to remedy truck drivers’ work conditions. The participants viewed their lifestyle as unhealthy and were aware of possible consequences. They expressed the need and wish to change their lifestyle, yet perceived it as an inherent, unavoidable outcome of their occupation. Current health improvement initiatives were not always aligned with their working conditions. The participants were generally willing to use mobile health technologies such as wearable devices, as a preventive measure to avoid prospect morbidity, particularly cardiovascular diseases. They were ambivalent about privacy and the risk of their employer’s monitoring their clinical data if such devices were to be used at work. Such technologies should be tailored to the particular needs and work conditions of truck drivers, and if found useful, could be later on extended to other types of shift workers. Further research on the acceptances and benefits of the health technology services will be needed once it is being offered and used by the drivers.

## Abbreviations

EIT, European Institute of Innovation and Technology; HGV, heavy goods vehicle; UK, United Kingdom.

## Appendix 1: Focus group schedule

### Warm up questions (not recorded)

W1. Tell me about your job; what does a regular working day look like?

W2. What do you like most about your job, what is the most interesting part?

### Discussion Theme A: Prior experience with health technology

A1. Have you been using any of them before or not?

A2. If yes, give examples of which one you used and how did you found them? Which elements or features did you like/dislike?

A3. If not, why? (Prompt: Lack of interest/knowledge?)

### Discussion Theme B. Motivations for using health technology

B1. At what situations did you/will you use these platforms?

B2. If you are currently using them, what motivates you to keep going back to these platforms? How are they useful for you?

B3. If you used some of these platforms before and stopped using them, why did you decide not to use them anymore?

### Discussion Theme C. Expectations from health technology platforms and wearable devices

C1. What are your expectations from the use of these devices/platforms?

C2. What are the main characteristics that you would like to see on these platforms and device?

C3. Will you use them in future? What would change your opinion?

### Discussion Theme D. Views on employers’ health promotion activities

D1. Do you think your employer is interested in your health?

D2. Why/why not?

D3. What need to happen in order for you to think that your employer is interested in your health?

### Summary/further thoughts

E1. Any further thoughts or comments you wish to make?
